# The Yield of Endoscopy and Histology in the Evaluation of Esophageal Dysphagia: Two Referral Centers’ Experiences

**DOI:** 10.3390/medicina57121336

**Published:** 2021-12-07

**Authors:** Amir Mari, Fadi Abu Baker, Helal Said Ahmad, Ali Omari, Yazed Jawabreh, Rand Abboud, Amir Shahin, Fahmi Shibli, Wisam Sbeit, Tawfik Khoury

**Affiliations:** 1 Gastroenterology and Endoscopy Units, The Nazareth Hospital, EMMS, Nazareth 16100, Israel; helal_sayid@yahoo.com; 2 Faculty of Medicine in the Galilee, Bar-Ilan University, Safed 1311502, Israel; amirs3@gmc.gov.il (A.S.); wisams@gmc.gov.il (W.S.); tawfikkhoury1@hotmail.com (T.K.); 3 Department of Gastroenterology, Hillel Yaffe Medical Center, Hadera 38100, Israel; fa_fd@hotmail.com; 4 Internal Medicine Department, The Nazareth Hospital, EMMS, Nazareth 16100, Israel; ali.omari.01993@gmail.com (A.O.); dr_jawabreh@yahoo.com (Y.J.); 5 Surgery Department, The Nazareth Hospital, EMMS, Nazareth 16100, Israel; rand.abboud@gmail.com; 6 Department of Gastroenterology, Galilee Medical Center, Nahariya 22100, Israel; 7 Department of Gastroenterology and Liver Disease, Emek Medical Center, Afula 1855701, Israel; fahmi_shibli@yahoo.ie; 8 Rappaport Faculty of Medicine, Technion—Israel Institute of Technology, Haifa 38100, Israel

**Keywords:** dysphagia, gastroscopy, diagnosis, eosinophilic esophagitis, endoscopic findings

## Abstract

*Background and Objectives*: The initial diagnostic test required to evaluate esophageal dysphagia is upper endoscopy (EGD) to assess the structure of the esophagus and the esophageo-gastric junction (EGJ). Taking biopsies during EGD has become a common practice in patients with dysphagia to rule out eosinophilic esophagitis (EoE). The aims of this study were to evaluate the endoscopic findings of patients who underwent EGD for esophageal dysphagia, to assess the rate of biopsy taking from the esophagus to diagnose/exclude EoE, and to report histology outcomes of these biopsies. *Materials and Methods*: This was a retrospective multicenter study that included individuals ≥18 years who underwent EGD due to esophageal dysphagia between the years 2015 and2020, (with no other alarm signs, such as weight loss, new iron deficiency anemia, and lymphadenopathy). We obtained data from patients’ electronic files. The endoscopy and histology findings were obtained from endoscopy reports saved in our electronic files. *Results*: A total of 209 patients were included in the study. The average age was 57.1 ± 17.1 years. The most common endoscopic findings were normal endoscopy in 76 patients (36.4%) and erosive esophagitis in 75 patients (35.9%). Barrett’s esophagus and esophageal malignancy were encountered in 11 patients (5.3%) and 2 patients (0.95%), respectively. Esophageal biopsies were taken in 50.2% of patients, and one patient had histological evidence of EoE (0.5%). On univariate analysis, there was a trend for association between proton pump inhibitors (PPIs) use and a normal EGD, but it was not statistically significant (OR 0.28, 95% CI 0.07–1.11, *p* = 0.07). *Conclusions*: Endoscopic findings were prevalent in dysphagia patients even when no other alarm symptoms exist. Neoplastic lesions and EOE were rare in our study.

## 1. Introduction

Dysphagia is a very common symptom that may affect up to 20% of patients in the primary care setting and represents a major cause for referral to gastroenterologists [1]. It is defined as the subjective sensation of difficulty swallowing during the passage of liquid or solid bolus, from the mouth to the stomach, or the awareness of an obstruction during the process of swallowing [2]. It can be further classified as oropharyngeal in which there is difficulty initiating a swallow or a choking sensation, coughing and possibly having nasal regurgitation during a swallow. Esophageal dysphagia, on the other hand, is a perceived sensation of food being stuck either in the throat or the lower chest [2].

The evaluation of dysphagia is a multistep pathway that includes a detailed medical history and physical examination, upper endoscopy imaging, and motility studies [2]. A careful history can differentiate between oro-pharyngeal, esophageal, and neuromuscular causes of dysphagia in up to 85% of patients [3]. 

The initial diagnostic modality required for a patient with esophageal dysphagia is upper endoscopy (EGD) to assess for structural abnormalities, such as rings, strictures and webs, inflammatory disorders (e.g., gastroesophageal reflux disease (GERD), eosinophilic esophagitis (EoE) and collagen disorders), and malignant or benign tumours [4]. Moreover, during endoscopy, some findings may suggest the presence of major motility disorders (i.e., achalasia and EGJ outflow obstruction). Suggestive findings of achalasia and EGJ outflow obstruction include fluid residue in the esophagus despite fasting, a dilated esophagus and signs of obstruction at the EGJ [4]. Notably, EoE is an inflammatory and allergic disease that represents an important cause of esophageal dysphagia and food impaction [5]. The most typical endoscopic findings of EoE include esophageal mucosal edema, ring formation, white exudates, furrows, and fibrotic strictures [6,7,8,9]. The diagnosis of EoE is confirmed by eosinophil-predominant inflammation (presence of ≥15 eosinophils/high-power field in at least one esophageal biopsy), which is nonresponsive to prolonged acid suppression with proton pump inhibitors [10]. The suggestive endoscopic findings of EoE present only in about half of cases, therefore, biopsy taking from all parts of the esophagus is recommended in all cases of dysphagia when EoE is suspected [10]. The primary aim of our study was to evaluate the endoscopic outcomes and findings of patients who underwent EGD for esophageal dysphagia, without other alarm signs, such as weight loss, new iron deficiency anemia, and lymphadenopathy. Moreover, our secondary aim was to assess the rate of biopsy taking from the esophagus to diagnose or exclude EoE, and, finally, we aimed to report histology outcomes of these biopsies.

## 2. Materials and Methods

This was a multicenter study utilizing patients’ data from two referral centers (Nazareth Hospital EMMS, Nazareth, Israel and Galilee Medical Center, Nahariya, Israel).

### 2.1. Patient’s Selection

Patients over the age of 18 years who underwent gastroscopy due to esophageal dysphagia between the years 2015 and 2020, (with no other alarm signs, such as weight loss, new iron deficiency anemia, and lymphadenopathy) were included. Patients with history of esophageal/gastric malignancy, surgery or radiation were excluded.

### 2.2. Data Management

We obtained data from patients’ electronic records. Demographics collected included age, gender, body mass index (BMI) and smoking status. Associated comorbidities of interest were hyperlipidemia, hypertension, diabetes mellitus, obesity chronic renal failure, congestive heart failure, GERD, asthma, allergy, celiac disease, inflammatory bowel disease and rheumatologic diseases, such as rheumatoid arthritis, ankylosing spondylitis, systemic lupus erythematosus (SLE) and scleroderma. Medications identified in our query were proton pump inhibitors (PPIs) and statins. The endoscopy and histology findings were obtained from endoscopy reports saved in our department’s electronic files. Moreover, the diagnosis of EoE was confirmed by taking multiple (more than 5) biopsies from the proximal and middle esophagus.

### 2.3. Statistical Analysis

Categorical variables will be presented as frequencies and percentages as they were analyzed by Chi-square test, while continuous variables were reported as mean ± SD using the two-sample *t*-test. Endoscopic findings were presented as frequencies. A univariate model analysis was performed to assess correlation between various demographic and clinical parameters and endoscopic findings, reporting odds ratio (OR) and confidence intervals (CI). Variables with *p* value less than 0.05 were considered statistically significant. Statistical analyses were performed by commercial software, Statistical Package for Social Science (SPSS version 24.0, IBM, Chicago, IL, USA).

## 3. Results

### 3.1. Demographics and Baseline Characteristics

A total of 209 patients were included in the study. The average age in the cohort was 57.1 ± 17.1 years. The male:female ratio was similar (51% vs. 49%). The rate of comorbidities including hyperlipidemia, hypertension, chronic renal failure, congestive heart failure, diabetes mellitus, ischemic heart disease, asthma, rheumatoid arthritis, ankylosing spondylitis, scleroderma, and systemic lupus erythematosus was 44.8%, 42.4%, 4.9%, 8.7%, 23.9%, 17%, 4.4%, 1.5%, 0.5%, 0.5% and 0.5%, respectively. Only 1.9% of patients had a history of atopy (Table 1).

### 3.2. Endoscopic Findings

The most common endoscopy findings were normal endoscopy in 76 patients (36.4%), followed by erosive esophagitis in 75 patients (35.9%), and hiatal hernia in 65 patients (31.1%). Barrett’s esophagus and esophageal malignancy were encountered in 11 patients (5.3%) and 2 patients (0.95%), respectively. Endoscopic features of EoE (esophageal furrows) were only seen in one patient (0.5%). Interestingly, of all the patients who had a biopsy taken (50.2%), only one had histological evidence of EoE (0.5%). Among patients with a normal endoscopy, 42 patients underwent esophageal manometry; of them, 10 patients (23.8%) were diagnosed with achalasia, 8 patients (19%) with ineffective esophageal motility disorder, 1 patient (2.4%) with hypomotility esophagus, 1 patient (2.4%) with Jackhammer esophagus, 1 patient (2.4%) with esophago-gastric outlet obstruction and 21 (50%) with a normal manometry study.

**Figure 1:**
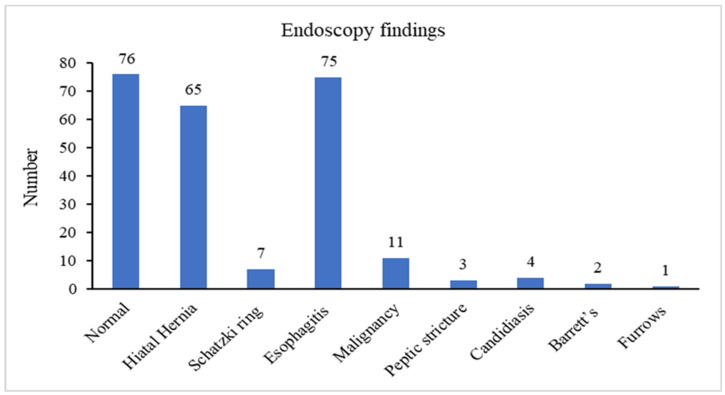
Endoscopic findings.

Figure 1 demonstrates the rate of endoscopic findings among the study cohort.

#### 3.2.1. Analysis of Normal Versus Pathological Endoscopic Findings

Seventy-four patients had normal gastroscopy (group A), as compared to 135 patients who had all pathological findings (including hiatal hernia, erosive esophagitis, Schatzki ring, malignancy, diverticulum, peptic stricture, Barrett’s, candidiasis and furrows) on gastroscopy (group B). The average age in group A was 53.9 years vs. 58.5 in group B (*p* = 0.07). Male gender accounted for 54.8% in group A vs. 44.6% in group B. Notably, smoking and hyperlipidemia were significantly more common in group B (44.7% and 50%), as compared to group A (31.1% and 35.6%, respectively, *p* = 0.02). There was no effect of the other assessed parameters (Table 2). 

#### 3.2.2. Analysis of Normal versus Barrett’s Esophagus and Neoplastic Endoscopic Findings

We compared normal gastroscopy (74 patients) with neoplastic endoscopic findings (13 patients), including esophageal malignancy and preneoplastic Barrett’s esophagus. We could not find any factor that was significantly associated with endoscopic esophageal findings, compared to patients with normal gastroscopy. On univariate analysis, there was a trend of association between chronic PPI use and a normal EGD, but it was not statistically significant (OR 0.28, 95% CI 0.07–1.11, *p* = 0.07) (Table 3). 

#### 3.2.3. Analysis of Pathological Non-Neoplastic vs. Neoplastic Endoscopic Findings

Comparing parameters that were associated with Barrett’s esophagus and neoplastic pathological findings on EGD, we found that only chronic PPI use for more than 3 months was significantly associated with a lower rate of neoplastic findings (*p* = 0.006). On univariate analysis, chronic PPI use was associated with a reduced risk of having endoscopic neoplastic findings (OR 0.18, 95% CI 0.05–0.7, *p* = 0.01). Notably, there were no associations of the other assessed parameters on the rate of the neoplastic endoscopic finding (Table 4).

## 4. Discussion

In the present study, we evaluated the yield of performing EGD with a biopsy of the esophagus while evaluating a patient with esophageal dysphagia without other alarm symptoms. According to this study result, one third of patients had a normal EGD whereas the majority of patients had benign endoscopic findings, and only 5% had neoplastic lesions. Various studies have assessed the endoscopic findings among esophageal dysphagia patients [1,11,12,13]. Esophageal strictures and Schatzki rings were the most prevalent causes of esophageal dysphagia in most reports, followed by malignant lesions. A study by Khan et al. showed that malignant strictures are the most common endoscopic pathology in dysphagia male patients [14]. GERD is a common gastrointestinal symptom among the general population, and the endoscopic manifestation of chronic GERD may vary from a normal esophagus to reflux esophagitis, strictures, Schatzki rings and Barrett’s esophagus [15,16,17]. Notably, GERD causes dysphagia even when esophageal mucosa is normal [18]. In our cohort, one third of patients had a normal gastroscopy, a finding that suggests non-erosive reflux disease as a possible etiology for dysphagia. Hiatal hernia is an arguable cause for dysphagia, however only the large hernias, of the complicated and para-esophageal types may contribute to dysphagia [19]. Hiatal hernia was common in our results, but the majority of these hernias were small and uncomplicated, therefore no mechanical mechanism is proposed to cause dysphagia, while the increased likelihood of GERD in these patients is more plausible. The presence of a malignant lesion is an important and serious cause of dysphagia [12,13,14,15]. In our cohort, only two patients have Barrett’s esophagus, and only 5% of patients had a malignant lesion as the cause of dysphagia. These results are not in line with the reported literature, which show a higher prevalence of Barrett’s esophagus with malignant lesions [20]. This discrepancy may be explained by the fact that our cohort was relatively young and have no alarm signs or symptoms except for dysphagia. Smoking was associated with pathological endoscopic findings and malignant lesions. This is in line with published literature where smoking is linked to erosive esophagitis, strictures, and Barrett’s esophagus, as well as esophageal and gastric malignancies [21,22,23]. Nonetheless, smoking was not associated with malignant lesions in our study. This could be explained by the retrospective nature of data collection that may involve an information bias, as well as the small sample size of the study cohort. EoE is an emerging disease with a growing prevalence and increasing awareness of this disorder [9,10]. Nonetheless, the reported prevalence of endoscopic findings suggestive of EoE, such as exudates, furrows and rings, were extremely low in our cohort. In about half of the cases, esophageal biopsies were taken to diagnose or rule out EoE. However, we believe that the endoscopic practice is promptly improving in this regard in concordance with the growing incidence and prevalence of EoE worldwide [9,10]. Importantly, the reported prevalence of EoE in dysphagia is 12–22% [24]. The prevalence of EoE increases among Caucasian males and with symptoms of atopy, such as asthma, eczema and allergies [25]. However, most studies are from western countries, and, to the best of our knowledge no reports from Middle Eastern countries are available for comparison and discussion. One more point, EoE differs between paediatric and adult populations by means of incidence, prevalence, clinical presentation and endoscopic findings. Our cohort included only adults where EoE was less prevalent, and this fact could partially explain the very low rate of EoE diagnosed. Our study had some limitations, including its retrospective nature where data including clinical and endoscopic findings were collected retrospectively. Another limitation is the fact that we were unable to extract the exact number of esophageal biopsies taken for the diagnosis of EoE as the endoscopist that performed the gastroscopy stated only if one or several biopsies were performed; however, we could extract that the endoscopist performed several biopsies from the proximal and middle esophagus to rule out EoE. Moreover, the relatively small sample size is another important limitation; finally, this study cohort is unrepresentative of the general Israeli population, since the study was conducted only in centers in the northern region in Israel. Our study has some strong points, such as accurate and homogenous data collection, since all data were saved in the hospitals’ electronic files. Moreover, detailed and accurate recording of data from endoscopic reports was performed. The most important strong point of the study is the strict patient selection and the fact that we have assessed only patients without alarm signs to evaluate the yield of gastroscopy on this population.

In conclusion, upper endoscopy is an important initial tool to evaluate esophageal dysphagia even in the absence of alarm signs since most patients in our cohort had findings, although benign. It is important to assess for EoE in clinical scenarios, and endoscopic and histologic features of the disease should be examined. More prospective, large multicenter studies are warranted to better address the yield of endoscopic findings when evaluating dysphagia, as well as to better estimate the epidemiology of EoE in the Israeli population.

## Figures and Tables

**Table 1:** Demographics and baseline characteristics.

Number of Patients	209
Average age (years), mean ± SD	57.1 ± 17.1
Gender, N (%)	
Male	107 (51)
Female	102 (49)
Hyperlipidemia	94 (44.8)
Hypertension	89 (42.4)
Chronic renal failure	10 (4.9)
Congestive heart failure	18 (8.7)
Diabetes mellitus	50 (23.9)
Ischemic heart disease	36 (17)
Asthma	9 (4.4)
Rheumatoid arthritis	3 (1.5)
Ankylosing spondylitis	1 (0.5)
Scleroderma	1 (0.5)
Systemic lupus erythematosus	1 (0.5)

**Table 2:** Analysis of parameters associated with any endoscopic pathological findings demonstrates descriptive statistics of parameters associated with pathological gastroscopy.

	Normal Gastroscopy	Any Pathological Findings	*p* Value
Number	74	135	−
Age, years (mean)	53.9	58.5	0.07
Male gender	33 (44.6)	74 (54.8)	0.08
Smoking	23 (31.1)	60 (44.7)	0.05
Hyperlipidemia	26 (35.6)	68 (50)	0.02
Hypertension	29 (39.7)	59 (43.9)	0.3
Chronic renal failure	3 (4.1)	7 (5.3)	0.3
Congestive heart failure	10 (13.5)	8 (6.1)	0.03
Diabetes mellitus	19 (25.7)	31 (22.9)	0.3
Ischemic heart disease	14 (18.9)	21 (15.9)	0.2
Asthma	3 (4.1)	6 (4.5)	0.4
Rheumatoid arthritis	2 (2.7)	1 (0.7)	0.1
Scleroderma	0	1 (0.7)	0.2
Systemic lupus erythematosus	0	1 (0.7)	0.2
Use of PPI > 3-month prior endoscopy	41 (55.9)	84 (62.4)	0.1
Use of statin > 3-month prior endoscopy	28 (37.5)	41 (30.6)	0.2

**Table 3:** Analysis of parameters associated with Barrett’s esophagus and neoplastic endoscopic findings, compared to the normal gastroscopy, demonstrates the descriptive statistics of the assessed parameters.

	Normal Gastroscopy	Neoplastic Findings	*p* Value
Number	74	13	−
Age, years (mean)	53.9	64	0.13
Male gender, N (%)	33 (44.6)	6 (46.2)	0.9
Smoking, N (%)	23 (31.1)	6 (46.2)	0.80
Hyperlipidemia, N (%)	26 (35.1)	3 (23.1)	0.39
Hypertension, N (%)	29 (39.2)	6 (46.2)	0.64
Chronic renal failure, N (%)	3 (4.1)	1 (7.7)	0.56
Congestive heart failure, N (%)	10 (13.5)	1 (7.7)	0.56
Diabetes mellitus, N (%)	19 (25.7)	1 (7.7)	0.16
Ischemic heart disease, N (%)	13 (17.6)	3 (23.1)	0.63
Asthma, N (%)	3 (4.1)	1 (7.7)	0.56
Rheumatoid arthritis, N (%)	2 (2.7)	0	−
Scleroderma, N (%)	0	0	−
Systemic lupus erythematosus, N (%)	0	0	−
Use of PPI > 3-month prior endoscopy, N (%)	38 (51.3)	3 (23.1)	0.059

**Table 4:** Analysis of parameters associated with Barrett’s esophagus and neoplastic endoscopic findings, compared to the pathological non-neoplastic findings.

Pathological Endoscopic Findings			
	Neoplastic Findings	Non-Neoplastic Findings	*p* Value
Number	13	121	−
Age, years (mean)	54.3	58.6	0.32
Male gender, N (%)	6 (46.2)	68 (56.2)	0.49
Smoking, N (%)	6 (46.2)	53 (43.8)	0.80
Hyperlipidemia, N (%)	3 (23.1)	62 (51.2)	0.053
Hypertension, N (%)	5 (38.5)	53 (43.8)	0.71
Chronic renal failure, N (%)	1 (7.7)	6 (4.9)	0.67
Congestive heart failure, N (%)	1 (7.7)	6 (4.9)	0.67
Diabetes mellitus, N (%)	1 (7.7)	29 (24)	0.18
Ischemic heart disease, N (%)	3 (23.1)	18 (14.9)	0.44
Asthma, N (%)	1 (7.7)	5 (4.1)	0.56
Rheumatoid arthritis, N (%)	0	1 (0.8)	−
Scleroderma, N (%)	0	1 (0.8)	−
Systemic lupus erythematosus, N (%)	0	1 (0.8)	−
Use of PPI > 3-month prior endoscopy, N (%)	3 (23.1)	75 (62)	0.006

## Abbreviations

EGD: upper endoscopy; EGJ: esophageo-gastric junction; EoE: eosinophilic esophagitis; PPI: proton pump inhibitors; GERD: gastroesophageal reflux disease; BMI: body mass index; SLE: systemic lupus erythematosus; OR: odds ratio; CI: confidence intervals.

## References

[1] Wilkins T., Gillies R.A., Thomas A.M., Wagner P.J. (2007). The prevalence of dysphagia in primary care patients: A HamesNet Research Network study. J. Am. Board. Fam. Med..

[2] Mari A., Sweis R. (2021). Assessment and management of dysphagia and achalasia. Clin. Med..

[3] Cook I.J. (2009). Oropharyngeal dysphagia. Gastroenterol. Clin. N. Am..

[4] Mari A., Patel K., Mahamid M., Khoury T., Pesce M. (2019). Achalasia: Insights into diagnostic and therapeutic advances for an ancient disease. Rambam Maimonides Med. J..

[5] Mari A., Tsoukali E., Yaccob A. (2020). eosinophilic esophagitis in adults: A concise overview of an evolving disease. Korean J. Fam. Med..

[6] Straumann A., Spichtin H.P., Bucher K.A., Heer P., Simon H.U. (2004). Eosinophilic esophagitis: Red on microscopy, white on endoscopy. Digestion.

[7] Peery A.F., Cao H., Dominik R., Shaheen N.J., Dellon E.S. (2011). Variable reliability of endoscopic findings with white-light and narrow-band imaging for patients with suspected eosinophilic esophagitis. Clin. Gastroenterol. Hepatol..

[8] Kim H.P., Vance R.B., Shaheen N.J., Dellon E.S. (2012). The prevalence and diagnostic utility of endoscopic features of eosinophilic esophagitis: A meta-analysis. Clin. Gastroenterol. Hepatol..

[9] Lucendo A.J., Molina-Infante J., Arias A., von Arnim U., Bredenoord A.J., Bussmann C., Amil Dias J., Bove M., Gonzalez-Cervera J., Larsson H. (2017). Guidelines on eosinophilic esophagitis: Evidence-based statements and recommendations for diagnosis and management in children and adults. United Eur. Gastroenterol. J..

[10] Mari A., Abu Baker F., Mahamid M., Khoury T., Sbeit W., Pellicano R. (2020). Eosinophilic esophagitis: Pitfalls and controversies in diagnosis and management. Minerva Med..

[11] Lind C.D. (2003). Dysphagia: Evaluation and treatment. Gastroenterol. Clin. N. Am..

[12] Richter J.E. (1998). Practical approach to the diagnosis and treatment of esophageal dysphagia. Compr. Ther..

[13] Krishnamurthy C., Hilden K., Peterson K.A., Mattek N., Adler D.G., Fang J.C. (2012). Endoscopic findings in patients presenting with dysphagia: Analysis of a national endoscopy database. Dysphagia.

[14] Khan A.N., Said K., Ahmad M., Ali K., Hidayat R., Latif H. (2014). Endoscopic findings in patients presenting with oesophageal dysphagia. J. Ayub Med. Coll. Abbottabad.

[15] Agreus L., Svardsudd K., Talley N.J., Jones M.P., Tibblin G. (2001). Natural history of gastroesophageal reflux disease and functional abdominal disorders: A population-based study. Am. J. Gastroenterol..

[16] Velanovich V., Karmy-Jones R. (1998). Measuring gastroesophageal reflux disease: Relationship between the health-related quality of life score and physiologic parameters. Am. Surg..

[17] El-Serag H.B., Sweet S., Winchester C.C., Dent J. (2014). Update on the epidemiology of gastro-oesophageal reflux disease: A systematic review. Gut.

[18] Gyawali C.P., Kahrilas P.J., Savarino E., Zerbib F., Mion F., Smout A., Vaezi M., Sifrim D., Fox M.R., Vela M.F. (2018). Modern diagnosis of GERD: The Lyon Consensus. Gut.

[19] Philpott H., Sweis R. (2017). Hiatus hernia as a cause of dysphagia. Curr. Gastroenterol. Rep..

[20] Layke J.C., Lopez P.P. (2006). Esophageal cancer: A review and update. Am. Fam. Physician.

[21] Kim B.J., Cheon W.S., Oh H.C., Kim J.W., Park J.D., Kim J.G. (2011). Prevalence and risk factor of erosive esophagitis observed in Korean National Cancer Screening Program. J. Korean Med. Sci..

[22] Lee D., Lee K.J., Kim K.M., Lim S.K. (2013). Prevalence of asymptomatic erosive esophagitis and factors associated with symptom presentation of erosive esophagitis. Scand. J. Gastroenterol..

[23] Matsuzaki J., Suzuki H., Kobayakawa M., Inadomi J.M., Takayama M., Makino K., Iwao Y., Sugino Y., Kanai T. (2015). Association of visceral fat area, smoking, and alcohol consumption with reflux esophagitis and Barrett’s esophagus in Japan. PLoS ONE.

[24] Dellon E.S., Hirano I. (2018). Epidemiology and natural history of eosinophilic esophagitis. Gastroenterology.

[25] Kumar S., Choi S., Gupta S.K. (2019). Eosinophilic esophagitis—A primer for otolaryngologists. JAMA Otolaryngol. Head Neck Surg..

